# Chorionic Gonadotropin and Its Receptor Are Both Expressed in Human Retina, Possible Implications in Normal and Pathological Conditions

**DOI:** 10.1371/journal.pone.0052567

**Published:** 2012-12-19

**Authors:** Sladjana Dukic-Stefanovic, Jan Walther, Sebastian Wosch, Gerolf Zimmermann, Peter Wiedemann, Henry Alexander, Thomas Claudepierre

**Affiliations:** 1 Department of Ophthalmology, Faculty of Medicine, University of Leipzig, Leipzig, Germany; 2 Department of Obstetrics and Gynecology, Division of Human Reproduction and Endocrinology, University of Leipzig, Leipzig, Germany; Institut de la Vision, France

## Abstract

Extra-gonadal role of gonadotropins has been re-evaluated over the last 20 years. In addition to pituitary secretion of luteinizing hormone (LH) and follicle stimulating hormone (FSH), the CNS has been clearly identified as a source of hCG acting locally to influence behaviour. Here we demonstrated that human retina is producing this gonadotropin that acts as a neuroactive molecule. Müller glial and retinal pigmented epithelial (RPE) cells are producing hCG that may affects neighbour cells expressing its receptor, namely cone photoreceptors. It was previously described that amacrine and retinal ganglion (RGC) cells are targets of the gonadotropin releasing hormone that control the secretion of all gonadotropins. Therefore our findings suggest that a complex neuroendocrine circuit exists in the retina, involving hCG secreting cells (glial and RPE), hCG targets (photoreceptors) and hCG-release controlling cells (amacrine and RGC). The exact physiological functions of this circuit have still to be identified, but the proliferation of photoreceptor-derived tumor induced by hCG demonstrated the need to control this neuroendocrine loop.

## Introduction

Human chorionic gonadotropin (hCG) is the hormone of pregnancy produced by the trophoblast shortly after implantation to maintain the steroid secretion necessary for the proper development of the embryo. hCG is a 38 kDa glycoprotein, composed of a 14, 9 kDa α-subunit and a 23 kDa β subunit. The α subunit of hCG is identical to α subunits of other human glycoproteins: luteinizing hormone (LH), follicle stimulating hormone (FSH), and the thyroid stimulating hormone (TSH); the β subunit therefore provides hormonal specificity [1]. In the past decades, aside from its role in pregnancy, several reports showed that hCG together with LH elicit multiple effects in the central nervous system [2]. Many of the behavioral changes induced by hCG injection in rats parallel those observed in pregnant women [3] and some of these behavioral effects are correlated with changes of eicosanoid metabolism induced by LH and hCG in the brain [4]. Administration of gonadotropins can induce appetite loss, facilitation of extinction of the conditioned avoidance response, decreased exploratory activity, and decreased electrical activity of the brain [5]–[8]. In addition, trophic effects of hCG have been identified during development, neuroregenerative processes and tumorigenesis in CNS. Culturing fetal brain neurons, in the presence of highly purified hCG, resulted in a dose-dependent increase of survival and of neurite outgrowth [9]. Treatment of rats with hCG after a complete transection of the spinal cord induced the presence of nerve fibers in the bridging tissue suggesting that hCG might be useful in functional recovery for patients with paraplegia [10]. Together with nerve growth factor, LH and hCG are member of the cysteine*-*knot growth-factor family; therefore observed neurotrophic effects might be linked to this specific structural motif [11]. Also aberrant hCG secretion is a hallmark of various cancer types including intracranial germinomas linked to endocrine abnormalities [12]. Cancers expressing hCG/subunits have poor prognosis that might be linked to the autocrine effect of hCG as growth promoter [13]–[15].

LH/hCG actions in the CNS is mediated by the cyclic AMP/PKA signaling cascade as treatment with LH/hCG increased cyclic AMP and PKA activity in immortalized neurons and specific pathway inhibitor abolished this effect [16], [17]. Biological activity of pituitary hCG was found 50% as active as hCG purified from the urine of pregnant women in cyclic AMP assays [18]. LH and hCG signal through a common G-protein-coupled receptor [19]. Although LH/hCG receptor (LHR) has been thought to be restricted to ovaries and testis, several evidence underlines its expression in non-gonadal tissue, including CNS [9], [20], [21]. There it may be involved in neuronal development and differentiation, sleep-wake activity, and regulatory feedback mechanism of gonadotropin-releasing hormone (GnRH) and LH synthesis in the hypothalamus and pituitary gland respectively [4], [8], [22], [23]. In addition, LHR expression has been identified in sensory systems [24], and especially, within the retina [25]. In this CNS tissue, LHR transcripts were found in higher density in the outer retina compared to the inner retina and were absent from the pigmented epithelium. Binding experiments using radio-labeled ^125^I-CG confirmed the presence of the receptor protein in the photoreceptor layer that was localized around the cell soma rather than outer segments [25]. However, up to now it was unclear if retinal LHR would be responsive to the circulating hormone as the LH/CG has been shown to cross the blood-brain barrier [8], or to a local source as there is no direct evidence of gonadotropin secretion within retina. Here we identified the expression and localization of hCG in the human retina at the messenger and protein level and compare it to LHR. We demonstrate that hCG was expressed in non-pregnant adult retina in human at a relatively low level compared to post-partum placental expression. This hormone was however clearly expressed by the major glial cells of the retina, the Müller glial cells (MGC) together with the retinal pigmented epithelium (RPE). Its receptor was broadly distributed with a predominant cone photoreceptor expression. In addition, we found LHR expressed at the surface of human retinoblastoma cell lines and demonstrated the positive action of hCG on the proliferation of these cancer cells derived from cone photoreceptors, an effect that was inhibit by anti-LHR co-treatment. All together our data suggest that hCG should not only be consider as the hormone of pregnancy but may also act as a neuroactive molecule in the normal and pathological physiology of the retina.

## Results

### hCG-beta is Expressed in the Human Retina

**Figure 1:**
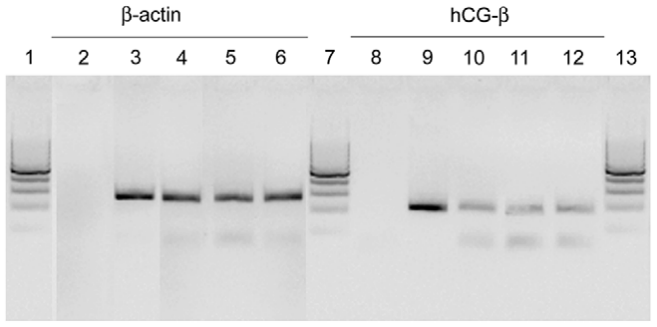
hCG-β mRNA expression in human retina. Expression of housekeeping beta-actin was comparable between samples (3–6). Expression of hCG-**β** was detected in all tested samples (9–12) with the exception of negative control (8). hCG-**β** mRNA was found in whole human retina (10), Müller glia cell line (11) and RPE cells (12). β-hCG expression was significantly lower than in human placenta (9). Amplification products were generated at the expected size (196 bp for hCG-**β** using primers GTCAACACCACCATCTGTGC and GGCAGAGTGCACATTGACAG, NM_000737). 1, 7, 13: DNA ladder (100 bp); 2–3: Housekeeping cDNA amplification (beta actin); 8–12: hCG-**β** cDNA amplification; 2, 8: negative control; 3, 9: Human placenta; 4, 10: Human retina; 5, 11: Immortalized human Müller glia cell line (MIO-M1); 6, 12: Primary human retinal pigmented epithelial (RPE) cells.

Messenger for beta hCG chain (hCG-**β**) was found in the placenta and in retina from donors (Figure 1). Amplification products were generated at the expected size (196 bp for hCG-**β**) but relative expression was strongly reduced in the retina compared to the placenta. ΔCT in retina was found to be 9.91+/−0.34 while it was equal to −3.19+/−0.28 in placenta (n = 8 for both tissues). Relative expression of hCG-**β** was therefore 8795.19 fold higher in the placenta compared to retina. The low retinal expression of hCG-**β** (∼0, 01% of the placental one) was however specific as we found it also in human glial cell lines MIO-M1 and primary human RPE cells (Figure 1, samples 11 and 12), suggesting that glial and RPE cells were a source of hCG-**β** within the retina.

**Figure 2:**
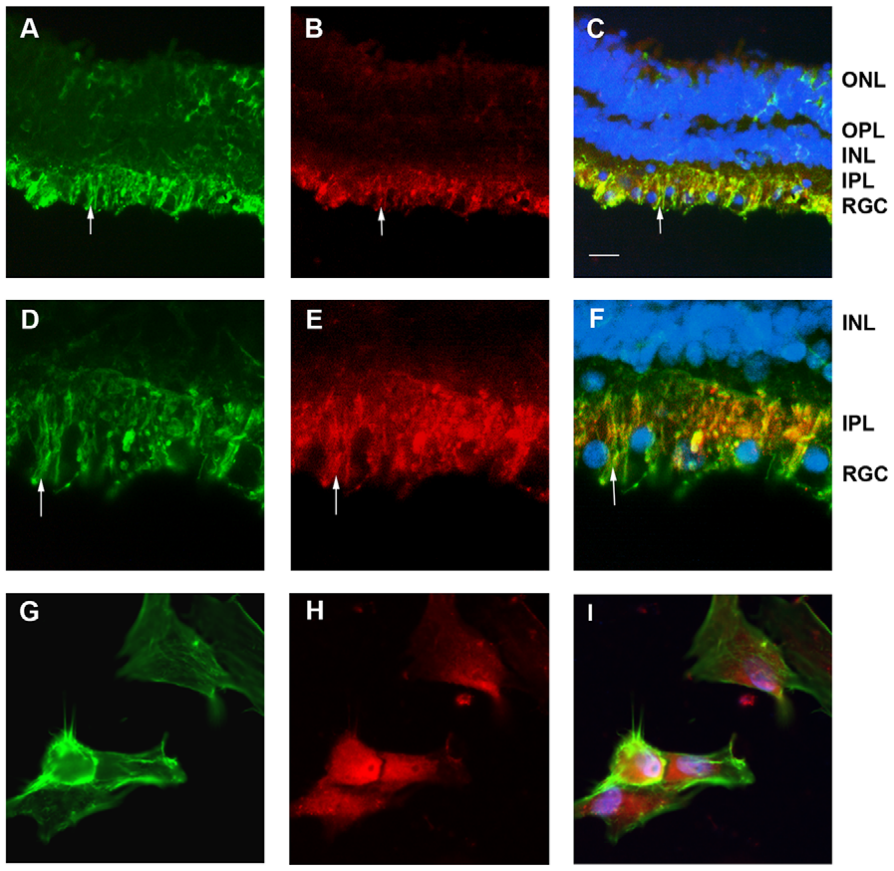
hCG-β localization in human retina. Vimentin labeled Muller glial cell of the retina mainly in the GCL and IPL (A, C, D, F). Müller glia cell processes are labeled with vimentin (A, D) and hCG-**β** (B, E) between the ganglion nucleus that are labeled in blue in the GCL (C, F). Arrow (A–F) depicts glia cell endfeet that is double labeled with hCG-**β** (C, F). Diffuse staining against hCG-**β** suggests a secretion of hCG in the retina (B, E). **hCG-β localization in human RPE**. Primary RPE cells kept in culture were stained with phalloidin (G) and hCG-**β** (H), Merged image (I) revealed the expression of this gonadotropin in the RPE cells. Scale bar is 30 µm for A–C 15 µm for D–F and 10 µm for G–I. A, D: Mouse monoclonal anti vimentin (Abcam ref# ab28028) use 1/100 in PBS, 0.1% BSA, 0.01% triton x100; B, E, H, I: Rabbit polyclonal anti hCG-**β** (Abcam ref# ab9376) use 1/100 in PBS, 0.1% BSA, 0.01% triton x100; G, I : Phalloidin-488 (Millipore ref# A12379) use at 1/50 in PBS; C, F, I : Merged image, with blue channel corresponding to DAPI nucleus staining used at 1/1000. ONL = Outer nuclear layer, OPL = outer plexiform layer, INL = Inner nuclear layer, IPL = Inner plexiform layer, GCL = Ganglion cell layer.

Using an antibody raised against the hCG-**β** we detected a radial signal throughout the retinal thickness (Figure 2 B) with an increasing intensity in the inner part of the retina (inner plexiform and ganglion cell layers, Figure 2 E). Such distribution was found similar to the Müller glial cell marker, vimentin (Figure 2 A, D). Merged image confirmed the colocalization and the glia expression of hCG-**β**. Magnification of the inner retina depicted hCG-**β** localization in the Müller glial cell endfeet (arrow in Figure 2 D–F) that surrounds the RGC soma visualized in blue via DAPI staining. Immunohistochemical stainings support the PCR results suggesting that Müller glial cells could be a source of hCG in the retina. Due to the preparation method, human retinal sections were devoid of RPE cells and we could not confirm here that this cell type played also a role in hCG-**β** secretion in vivo. However, in vitro, primary RPE cells were also labeled by the anti-hCG-**β** antibody (Figure 2 G–I). Cells were obtained from donors as described previously [26] and stained for actin filament (Figure 2 G) and hCG (Figure 2 H). On the merged image, (Figure 2 I), the gonadotropin was localized in the whole cell soma, suggesting a cytoplasmic location.

### Receptor for hCG within the Retina

**Figure 3:**
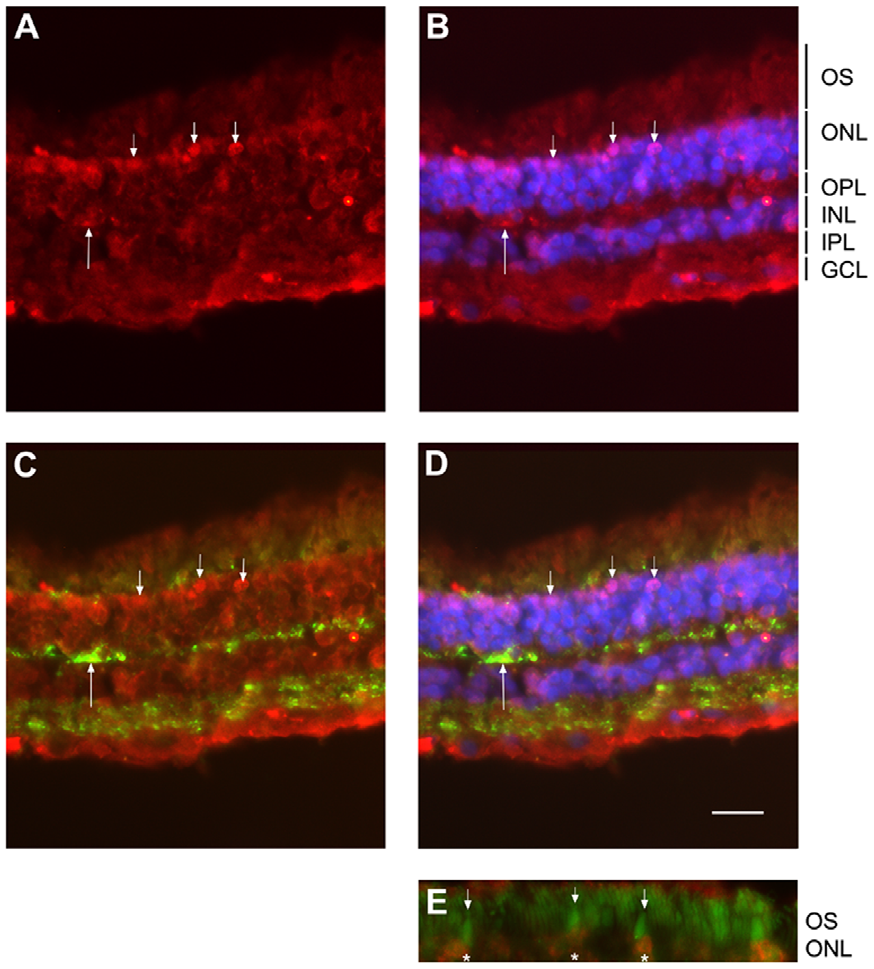
LHR localization in human retina. LHR was found expressed throughout the human retina with an enhanced signal in the photoreceptor layer (ONL) especially in the most superficial layer (small arrows in A–D), signal was found around cell soma in the ONL but also rarely in the OPL (long arrow in A–C) and in the INL. In addition a diffuse signal was found in the basal part of the GCL. The DAPI co-staining confirmed that photoreceptor in the most outer layer were labeled (small arrows in B). Synaptophysin labeled presynaptic structures in the OPL and IPL synaptic layers (C, D). Large stained structures in the OPL corresponded to a cone terminals (long arrow in C) that was also found labeled with LHR antibody (long arrow in A–C) suggesting that stained neurons in the ONL with anti-LHR may correspond to cone photoreceptors. Photoreceptor labeled with LHR antibody in the most superficial layer of the ONL (asterisks in E) exhibit outer segment with a cone shape as stained with anti CD133 antibody (small arrow in E) thus confirming the cone identity of the LHR-positive photoreceptors. Scale bar is 30 µm for A–C and 15 µm for E. OS = outer segment, ONL = outer nuclear layer, OPL = outer plexiform layer, INL = inner nuclear layer, IPL = inner plexiform layer, GCL = ganglion cell layer. A–E : Red channel, rabbit polyclonal anti LHR (Santa Cruz biotechnology ref# ab25828) use 1/100 in PBS, 0.1% BSA, 0.01% triton x100; B : Merged image, with blue channel corresponding to DAPI nucleus staining used at 1/1000; C : Merged image, with synaptophysin (green channel) using mouse monoclonal anti synaptophysin (Millipore ref# MAB368) at1/100 in PBS, 0.1% BSA, 0.01% triton x100; D: Merged image with synaptophysin (green) and DAPI (blue); E : Merged image, with green channel corresponding to CD133 staining using mouse monoclonal anti CD133 (eBioscience ref #14–1331) at 1/1000in PBS, 0.1% BSA, 0.01% triton x100.

The receptor for hCG was identified within the human retina (Figure 3). Using a rabbit raised against the common receptor for luteinizing and choriogonadotropin hormones (LHR) we observed a diffused staining throughout the retina with an increased specific signal in cells located in the first rows of the photoreceptor layer and in structure in the outer plexiform layer, corresponding to the synaptic connection of the photoreceptors with the inner retina. Using an anti-synaptophysin to reveal synapses in the plexiform layer we observed a colocalization of the staining in large synaptic structures corresponding morphologically to cone terminals (arrow in Figure 3 A–C). When using an anti-CD133 antibody to reveal the outer segments, we observed that in the first row of cells, the one labeled with LHR antibody are directly apposed to the cone outer segment (arrowheads in Figure 3 D). Altogether those observations suggest that cone photoreceptors strongly express LHR at their membrane.

**Figure 4:**
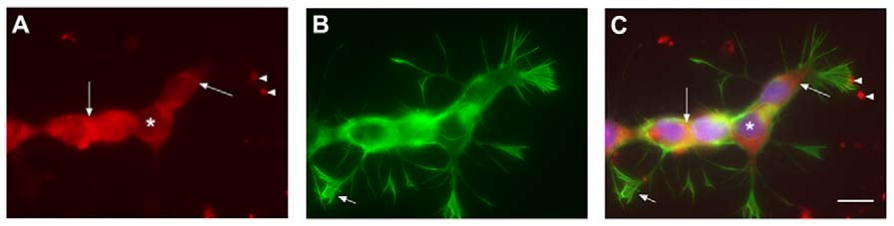
Human retinoblastoma cells express LHR. Gonadotropin receptor LHR was found expressed at retinoblastoma surface. Punctuate signal was enhance and around cell soma and at cell contact (arrows in A, C) while nucleus was not stained (asterisk in A, C) Fillopodia and lamelipodia extensions of retinoblastoma, heavily stained with phalloidin (B) were not labeled for LHR. Clusters of LHR-positive structures may account for membrane pieces of dead/detached retinoblastoma cells in the culture (arrowhead in A, C). Scale bar 5 µm. A: Rabbit polyclonal anti LHR (Santa Cruz biotechnology ref# ab25828) use 1/100 in PBS, 0.1% BSA, 0.01% triton x100; B : Phalloidin-488 (Millipore ref# A12379) use at 1/50 in PBS; C : Merged image, with blue channel corresponding to DAPI nucleus staining used at 1/1000.

Moreover, Y79 Retinoblastoma cells line, a cone precursor tumor [27] was tested for LHR expression and we found the hCG receptor concentrated at the cancer cell membrane (Figure 4).

### Western Blot Analysis

**Figure 5:**
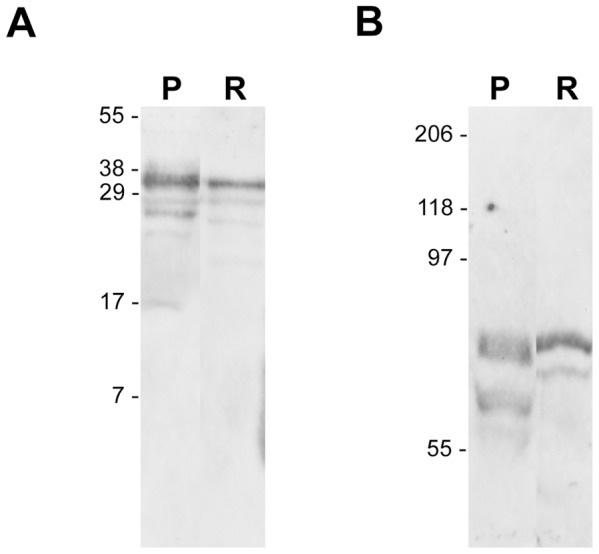
Western blot analysis of hCG and its receptor in retina. A) Both placental and retinal extracts expressed hCG as detected on western blot using an antibody recognizing the whole hormone (Abcam ab 54410). Using 0.1 and 30 µg of protein extract from placenta (P) and Retina (R) respectively a major band is found in both tissues at an apparent molecular weight of 37 kDa corresponding to the heterodimer predicted sized. Smaller bands are seen in the placenta (17, 27, 29 kDa) and in the retina (20, 27, 29 kDa). **B)** LHR was detected in 10 µg of placental and retinal extract loaded on a 7.5% SDS gel. Major upper band at 85 kDa corresponds to the glycosylated form of the receptor whereas lower bands observes in placenta at 70 kDa (doublet) and in retina at 80 kDa may account for partially or un-glycosylated products.

Using an antibody raised against the whole hormone we detected the whole hCG in placental and retinal extract (Figure 5 A). We loaded 100 ng of protein of the placental extract and 30 µg of the retinal extract on an 18% SDS-PAGE gel and detected a major band at 37 kDa and smaller products ranging from 17 to 30 kDa in those tissues, corresponding to individual chains and/or degradation products. In addition we detected LHR on both placenta and retina when loading 10 µg of protein extracts on a 7.5% SDS-PAGE (Figure 5 B). Migration profile gave a apparent molecular mass of 85 kDa for the major band detected in both tissue. Smaller products have been also detected with an apparent molecular mass of 70 kDa (doublet) and 80 kDa in the placental and retinal extracts respectively. The major bands may correspond to the glycosylated form of the receptor while smaller products may correspond to partially or un-glycosylated forms of the receptor [28]. We did not detected larger oligomer/aggregate of LHR. Our results showed that both tissues expressed similar level of this glycosylated gonadotropin receptor but retina produced only small amounts of hCG compared to placenta.

### hCG and Retinoblastoma Proliferation

**Figure 6:**
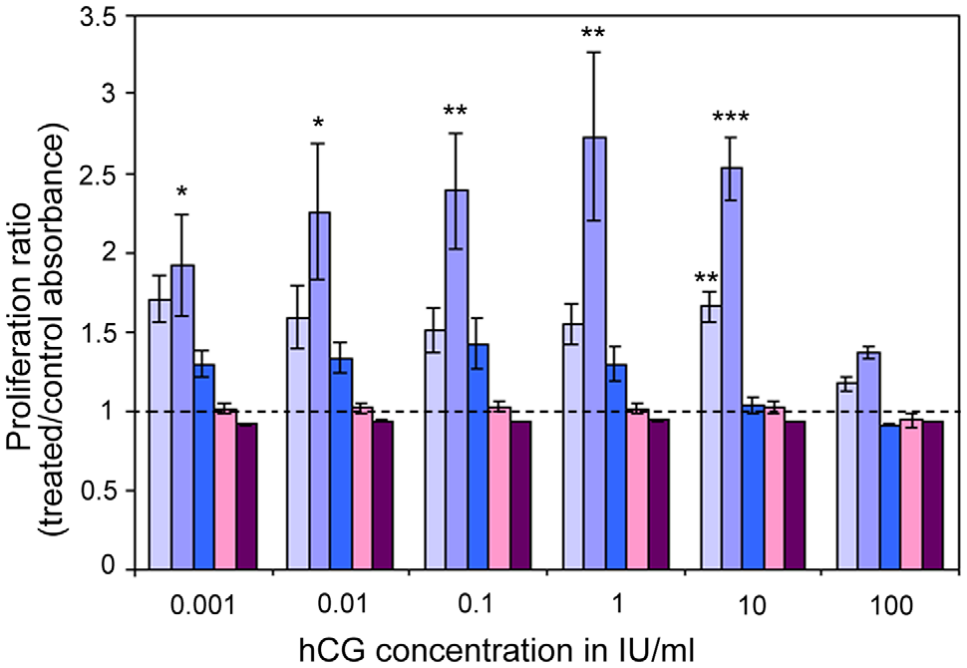
hCG treatment induces retinoblastoma proliferation. Y79 retinoblastoma cells were treated with increasing concentration of hCG (0,001 to 100 IU/ml) for 60, 120 and 240 hours (light, middle and deep blue bars). Using wst-1 test, we measured increase of proliferation in retinoblastoma treated with hCG by calculating the absorbance ratio of treated vs untreated cells at these 3 time points. In addition, blocking antibody raised against a signaling extracellular domain of LHR was added during the same period (light and deep purple bars). We observed that hCG treatment at all concentrations, but 100 IU/ml, induced a relative increase in proliferation after 60 and 120 hours. This increase reached significance after 60 h (light blue bars) only for the 10 IU/ml condition. After 120 h (middle blue), all concentrations, except the highest, significantly increased the retinoblastoma cell proliferation. After 240 h (deep blue bars), no significant effect of hCG was observed. LHR antibody inhibits this effect for all concentrations tested after 60 (light purple bars) and 120 hours (deep purple bars).

When treated with hCG, Y79 showed an increased proliferation as revealed by wst1 assay (Figure 6). After 60 hours of treatment (light blue bars), the effect was statistically significant with 10 mU/ml of hCG only, while after 120 hours (middle blue bars), the proliferation was significantly increased with hCG concentration ranging from 0,1 mIU/ml to 10 IU/ml. After 240 hours hCG treatment had no statistical effect on retinoblastoma proliferation as values were comparable to the control situation (deep blue bars). This might be due to the half-life of the hormone and/or its dissociation into individual α and β chains that are known to exert opposite biologic activity [29]. Interestingly, treatment with 100 IU/ml of hCG did not stimulate retinoblastoma cell proliferation. With this concentration, wst1 values at all three time points were comparable to the control situation as absorbance ratio was close to 1 (horizontal dashed line). The specificity of the hCG effect on retinoblastoma cell proliferation was confirmed by the co-treatment with 2 ng/ml anti-LHR antibody raised against an extracellular domain of human LHR involved in signalization. Using anti-LHR, the proliferation induced by hCG was completely abolished and values were similar to the untreated Y79 cells after 60 (light purple bars) and 120 hours of co-treatments (deep purple bars), while another antibody, raised against beta-clusterin, did not compromised the effect of hCG treatment (data not shown).

## Materials and Methods

### Human Tissues

The use of human material was approved by the Ethics Committee of the University of Leipzig, and was performed according to the declaration of Helsinki. All tissues were obtained after receiving written informed consent from the patients or their legal representatives. Copies of the consents were sent to the Ethics Commitee of the University for archiving. Eyes were obtained from adult post-mortem male donors without reported eye diseases within 48 h of death. Postpartum placentas were obtained from the Gynecology clinic in Leipzig.

### Human Retinoblastoma Culture

For immunocytochemistry, Y79 human retinoblastoma cell line (# HTB-18, ATCC, Manassas, VA) were cultured on BD Falcon 8 wells glass chambers slides (#354108, Becton-Dickinson, Franklin Lakes, NJ) previously coated with 10 µg/ml of laminin-1 (L2020, Sigma, St. Louis, MO). Cells were seed at 100.000/cm2 and cultured in DMEM Glutamax medium (#21885, InVitrogen) supplemented with 250 µM dibutyryl cyclic AMP (Sigma) 1000 U/ml penicillin and streptavidin (#15140-122, InVitrogen) and 10% fetal calf serum (#16000-044, InVitrogen). Those conditions allowed the adherence of the retinoblastoma cell lines of the glass surface as described elsewhere [30]. For proliferation assay, 20.000 of Y79 cells were seeded per well on flat bottom 96 well plate (Greiner Bio-One, Flickenhausen Germany).

### Immunohisto- and Immunocytochemistry

LHR and hCG-**β** were localized in human retina obtained from adult male donors with no tumor history. Retina was dissected from the eye and fixed during 15 minutes in a 4% formaldehyde solution in PBS, followed by increased sucrose gradients (10, 20 and 30% in PBS). Retinae were embedded in TissueTek cryomedium (Miles Eckhart, USA). Cryosections (10 µm) were collected on Superfrost Plus (Thermo Fischer scientific) glass slides. For retinoblastoma, cells were fixed in the same conditions before immunostaining process. Aspecific epitopes were blocked and cells were permeabilized using a solution of 30% casblock (InVitrogen) and 0.2% Triton X100 in PBS for 30 minutes at RT. Primary rabbit anti-hCG-**β** (# ab9376, Abcam) and rabbit anti-LHR (sc-25828,. Santa Cruz) antibodies were both used on retina sections overnight at 4°C, diluted 1/100 in PBS, casblock 3%, 0.02% triton X100. Markers of retinal cell types were used to identify cells expressing hCG-**β** and LHR: mouse anti vimentin (# ab28028, Abcam) and mouse anti synaptophysin (# MAB368, Millipore) were used at 1/100 while mouse anti CD133 (#14–1331, EBioscience San Diego, CA) was used at 1/1000. Corresponding secondary antibodies coupled to Alexa 488 and Alexa 555 for anti-goat were selected from InVitrogen and used at 1/500 for one hour at RT. Finally, a DAPI (InVitrogen) nuclear staining was achieved in the last washing steps. In Y79, actin filament were revealed using phalloidin coupled to Alexa 488 (#A12379 InVitrogen) and used at 1/50. Slides were mounted with Fluoromount-G (EMS, Hatfield, USA), observed using a Zeiss Axioplan 2 fluorescent microscope and picture taken with a Axiocam MRc5 digital camera coupled to Axiovison 4.6 software (Carl Zeiss). Control assays omitting primary antibodies confirmed the specificity of the staining (data not shown).

### Western Blot

Human tissues were homogenized in 10 volumes extraction buffer containing 150 mM NaCl, 10 mM EDTA, 1% protease inhibitor (Sigma-Aldrich), 1 mM phenylmethylsulfonyl fluoride (Sigma-Aldrich) and 25 mM Tris–HCl at pH 7.4. After centrifugation at 1000×*g* for 10 min, protein concentration was determined by BCA assay (Pierce/Thermo Fisher Scientific, Rockford, IL) following the manufacturer’s instructions. Protein extracts were resolved by SDS-PAGE gel electrophoresis and then transferred to reinforced nitrocellulose membranes (Optitran BA-S 85, Schleicher & Schuell BioScience, Dassel, Germany). The efficiency of the protein transfer was assessed by Ponceau S (Sigma-Aldrich) staining of membranes and Coomassie brilliant blue (Sigma-Aldrich) staining of blotted gels. Immunoblots were blocked with 1% bovine serum albumin (BSA, Sigma-Alrich) and 3% non-fat dry milk in phosphate-buffered saline (PBS; 137 mM NaCl, 2.68 mM KCl, 10 mM Na_2_HPO_4_, 1.76 mM KH_2_PO_4_, pH 7.4) and incubated overnight at 4°C with the following primary antibodies diluted in 0.5% Tween-20 in PBS: anti-hCG (1/500, rabbit polyclonal, ab54410, Abcam), anti-LHR (1/200, rabbit polyclonal, sc-25828, Santa Cruz), anti-GAPDH (1/5000, rabbit polyclonal, GTX100118, Gene Tex, Hsinchu, Taiwan). Thereafter, immunoblots were washed and incubated for 1 h at room temperature with horseradish peroxidase-conjugated secondary antibodies (#111-035-003, Jackson Immunologicals, West Grove, PA, USA) at 1/10000. Chemiluminescence was revealed by the Pierce ECL kit (#32106, Thermo Fisher Scientific) and detected using a Chemidoc XRS system (BioRad Hercules CA). Controls for the specificity of the signals were achieved by omitting the primary antibodies and by using prestained molecular weight marker, ranging from 7 to 209 kDa (# 161-0318, BioRad) to verify the correct migration of the reacting bands at their expected positions.

### hCG and Anti-LHR Treatments

1000 unit of highly purified hCG from pregnant urine (MBS173051, MyBioSource.com, San Diego, CA) were resuspend in 100 µl of distilled water and use to treat Y79 retinoblastoma cell at concentrations varying from 0,1 to 100 IU/ml, corresponding to a final molarity of 0,22 pM to 0,22 µM. In parallel LH/hCG receptor was blocked using LHR antibody (#sc-25828, Santa Cruz) developed in rabbit and raised against amino acids 28–77, mapping within an extracellular domain of LHR of human origin involved in signalization (http://www.uniprot.org/uniprot/P22888) [31]. LHR antibody was used at 2 ng/ml. Controls of the specificity of its action was achieve by treating Y79 with an antibody against clusterin-β (sc-13747, Santa Cruz), a rabbit polyclonal IgG, raised against an epitope near the N-terminus of clusterin-β of human origin and containing the same concentration of sodium azide, gelatin and immunoglobulin as the LHR antibody. Y79 cells were treated during 60, 120 and 240 hours and proliferation assay using tetrazolium salt wst-1 (#05015944001, Roche, Mannheim Germany) was performed by adding 1/10^th^ of wst-1 reagent to each well for 150 min (37°, 5% CO2). Resulting colorimetric reaction of the formazan dye generation was measured at 450 nm and 650 nm on a microplate reader (Spectramax 250, Molecular Devices, Sunnyvale, CA). The amount of formazan dye formed reflected the number of metabolically active cells in the culture and therefore, the measured absorbance directly correlated to the number of viable cells. Increase of absorbance at different time points reflects a proliferation. We measured the proliferation induced by treatments compared to the corresponding control in each individual experiment. Background value obtained for the medium alone was withdrawn from the resulting data and proliferation increase was reflected by the ratio of treated vs untreated cell absorbance after 60, 120 and 240 hours.

### Total RNA Extraction

Total RNA was extracted from RPE-cells by using the RNeasy Mini Kit (Qiagen, Hilden, Germany). The quality of the RNA was analyzed by agarose gel electrophoresis. The A_260_/A_280_ ratio of optical density was measured using the GeneQuantpro device (Pharmacia, Uppsala, Sweden), and was between 2.0 and 2.2 for all RNA samples, indicating sufficient quality. After treatment with DNase I (Roche, Mannheim, Germany), cDNA was synthesized from 1 µg total RNA using the RevertAid H Minus First Strand cDNA Synthesis kit (Fermentas, St. Leon-Roth, Germany).

### Real-time RT-PCR

The relative mRNA level of hCG-**β** in retina was determined in comparison to the placenta. Quantitative real-time RT-PCR was performed with the Single-Color Real-Time PCR Detection System (BioRad, Munich, Germany) using the following primer pairs sequence (forward/reverse) for beta actin (ACTB, NM_001101): 5′-ATGGCCACGGCTGCTTCCAGC-3′/5′-CATGGTGGTGCCGCCAGACA-3′ (237 bp); and for hCG-**β** (NM_000737): 5′-GTCAACACCACCATCTGTGC-3′/5′-GGCAGAGTGCACATTGACAG-3′ (196 bp). The PCR solution contained 1 µl cDNA, specific primer set (0.2 µM each) and 10 µl of a 2× mastermix (QuantiTect SYBR Green PCR Kit; Qiagen) in a final volume of 20 µl. The following conditions were used: initial denaturation and enzyme activation (one cycle at 95°C for 15 min); denaturation, amplification and quantification, 45 cycles at 95°C for 30 s, 58°C for 30 s, and 72°C for 1 min; melting curve, 55°C with the temperature gradually increased (0.5°C) up to 95°C. All PCR data were checked for homogeneity by dissociation curve analysis. Fluorescence changes were monitored after each cycle, *C*t (threshold cycle) values for amplification of hCG-**β** and β-actin mRNA were defined, and the level of hCG-**β** mRNA in each sample was standardized to the endogenous β-actin level. Comparable efficiencies for hCG-**β** and β-actin mRNA amplification were determined by analyzing serial cDNA dilutions. The changes in hCG-**β** mRNA expression were calculated according to the 2^−ΔΔCT^ method (CT, cycle threshold), with ΔCT = CT_hCGb_− CT_actb_ and ΔΔCT = ΔCT_retina_ − ΔCT_placenta_. The amplified samples were analyzed by standard agarose gel electrophoresis running samples from retina, placenta, retinal pigmented epithelial and Müller glial cells cultures. For this purpose primary human retinal pigmented epithelial cells and spontaneously immortalized human Müller cell line (MIO-M1) were cultured and prepared as described previously [26], [32].

### Data Analysis

Each experiment was at least repeated 3 times per conditions. Bar diagrams display the means of Y79 proliferation rate (± SEM). Comparisons between the means across all experimental conditions were made by analysis of variance (ANOVA). *P*<0.05 was considered statistically significant.

## Discussion

Here we demonstrated the existence of local source of hCG and the presence of its receptor within the human retina, a finding that widens the role of this gonadotropin within the CNS. It was previously known that a low level of hCG was secreted by the pituitary gland together with LH [33]. Secreted pituitary hCG has half of the biological activity of placental hCG, that has been shown to be 100 times more potent than LH [34]. When comparing the ratio of those two hormones present within the pituitary gland, despite its low level, hCG still has an average potency of one third of LH present [18]. The origin of this chemical difference is due to the multiple glycosylations of the hormone that make hCG a significant neuroactive molecule from the pituitary gland. In a similar manner, the low level of β-hCG within the retina when compared to its placental expression may not mandatory reflect an accessory role. Its biological activity may also be enhanced by specific glycosylations and by the topical release of the molecule within the retina, in close vicinity to its target. In mammals, the neuroendocrine control of gonadotropin hormones (LH, FSH and β-subunit of hCG) is mediated by the secretion of hypothalamic gonadotropin releasing hormone (GnRH-I), which activates a single receptor (GnRHR-I) located on pituitary gonadotrope cells. GnRHR-I has been found in different areas of the central nervous system as well as human placenta, endometrium, gonads, lymphocytes, breast, and prostate [35]–[38]. In addition, a second isoform (GnRH-II) acts both in the hypothalamus and other organ systems via the same receptor. Recently the retina of fishes and mammalian has been identified as a target for GnRH, expressing its receptor during development and in adulthood [39], which was specifically found in amacrine cells [40] and on some of retinal ganglion cell axons in the optic nerve [41]. Based on those observations it was suggested that, within the retina, GnRH may play a role in the control of seasonal and circadian rhythms in reproductive physiology. Our data are in line with those results and hypothesis, as we identified the expression of HCG and LHR receptor on glial and photoreceptor cells, respectively: Thus suggesting a complex mechanism for the regulation of gonadotropin release within the retina, which may achieve several physiological functions in the eye.

### Vision Changes Linked to Pituitary Condition

Change in vision linked to pituitary condition during normal and pathological pregnancies have been reported in the literature. A physiologic pituitary enlargement is a normal condition found in pregnant woman [42]. It usually disappears after delivery but may induce vision changes including reduced acuity and blurred vision [43]. However this condition can also be pathological due to pituitary tumor development, leading to vision loss and diplopia in both male and female [44]. A link with gonadotropin and its receptor we found expressed within the retina may be drawn due to the complications observed following GnRH injection to test the adenoma proliferation [45]. Authors have described few case of pituitary apoplexy, followed by partially reversible blindness after GnRH injection [46], [47].

### hCG and Vascularization

hCG is a key actor of placental vascularization [48] and hCG treatment was shown to promote growth of pericytes and vascular endothelial cell in vitro [49], suggesting a role of hGC and its receptor in angiogenesis. Interestingly, similar results were obtained on retinal perivascular and endothelial cells in culture [50]. In addition, as Müller cell processes surround the blood supply within the retina, hCG secreted by this glial cell may influence the perivascular structure in normal and pathological conditions.

### hCG and Ocular Pressure

It has been shown for many years that hCG injection lowers intraocular pressure (IOP) in rabbit [51], [52]. The mechanism of action was unknown and LHR was not yet identified within the retina, therefore some authors suggested that hCG action was due to a purity artifact as the results were not mimics by the LH [53]. However intraventricular, but not intravenous injection, of gonadotropin releasing hormone mimic the IOP decrease [54], demonstrating the specificity of hCG action in this mechanism. Interestingly pregnancy is usually associated with relatively lower IOP, but the mechanism of action is not yet identified [55], [56]. In women with preexisting glaucoma condition, IOP remain stable despite a reduction in the number of hypotensive agents during pregnancy [57]. Gonadotropin injection may therefore consist in a pressure lowering agent in the treatment of glaucomatous pathologies as suggested many years ago [58], [59]. Further study on the localization of LHR receptor within the structures involved in the regulation of ocular pressure (ciliary body, trabecular meshwork and vasculature) would help to decipher the cascade of events leading to IOP reduction.

### hCG, Photoreceptor Maturation and Circadian Clock

A previous study [25] and our present work suggest that photoreceptor cells, especially cone, express LHR and are therefore putative targets for hCG within the retina. In fish it has been shown that hCG treatment induced changes in the type of opsin synthesized by photoreceptor cells, resulting in a shift in their photosensibility [60], [61]. The cone localization of LHR suggests a direct effect of hCG and may reflect a role of gonadotropin in the differentiation/maturation of this specific cell type and a possible involvement of photoperiodic regulation for this neuroendocrine circuit.

### hCG and Retinoblastoma

We observed that hCG treatment increased, via LHR, the proliferation of Y79 retinoblastoma cell line in vitro. This may suggest a potential risk of gonadotropin use in women infertility treatment on the incidence of this malignant tumor occurrence. Retinoblastoma account for 11% of cancer cases in the first four years of life [62] and a link with in vitro fertilization has been pointed out by several authors [63]–[65]. However, other studies did not established a clear link with this specific type of cancer [66]–[68] even if the increased risk of pediatric cancers after infertility treatment have been globally demonstrated [69]. The discrepancy between the studies may be due to the low incidence of this disease (1 out of 15–20 000 children) leading to only few cases of retinoblastoma after infertility treatment. For instance, in the work of Foix-L’hélias (2012), only 8% of the retinoblastoma cases studied (20 out of 244) occurred in children whom mothers had undergone various infertility treatments [68]. Our finding suggests that future studies on the potential hazard of gonadotropins used for ovarian stimulation should take into account the exact protocol of the hormonal treatment (length, type and concentration of gonadotropins).

### Conclusion

As suggested by some authors [66], [70], [71] further evidences are needed before extragonadal expression of LH and hCG can be considered fully functionally significant, but present results together with previous studies in the literature [39], [40], [72], [73] suggest that visual processing may be influenced or regulated by gonadotropin reproductive hormones coming from the retina itself and acting locally as neuromodulators via LHR.
